# An NLP-Driven Framework for Automated Radiology–Pathology Concordance Assessment in Breast Biopsy

**DOI:** 10.3390/diagnostics16091249

**Published:** 2026-04-22

**Authors:** Emel Esmerer, Mehmet Ali Nazlı, Meryem Uzun-Per, Melike Gümüş Değidiben, Merve Söyleyici, Eren Tahir, Mert Bal

**Affiliations:** 1 Department of Radiology, Başaksehir Çam and Sakura City Hospital, Basaksehir, Istanbul 34480, Turkey; mnazli46@yahoo.com (M.A.N.); melikegcetin@icloud.com (M.G.D.); mervesoyleyici@hotmail.com (M.S.); 2 Department of Computer Engineering, Istanbul Health and Technology University, Zeytinburnu, Istanbul 34275, Turkey; meryem.uzunper@istun.edu.tr; 3 Data Science and Big Data Master’s Program, Institute of Science and Technology, Yildiz Technical University, Zeytinburnu, Istanbul 34220, Turkey; eren.tahir@std.yildiz.edu.tr; 4 Department of Mathematical Engineering, Yildiz Technical University, Davutpasa, Istanbul 34220, Turkey; mertbal@yildiz.edu.tr

**Keywords:** natural language processing, radiology–pathology concordance, breast biopsy, machine learning, artificial intelligence

## Abstract

**Background/Objectives**: To develop and assess the feasibility of a natural language processing (NLP) framework for automated assessment of radiology–pathology concordance in breast biopsy using machine learning-based analysis of unstructured reports. **Methods**: This retrospective study included 766 paired radiology and pathology reports from ultrasound- or mammography-guided breast biopsies (August 2020–May 2024). Reports underwent translation, normalization, tokenization, lemmatization, and synonym expansion, followed by structured encoding of BI-RADS and pathology categories. Three models were trained: a Decision Tree, a LightGBM classifier, and a fine-tuned BioBERT model. Concordance labels were defined by multidisciplinary consensus. Performance metrics included accuracy, sensitivity, specificity, F1-score, area under the curve (AUC), and Cohen’s kappa. SHapley Additive exPlanations (SHAP) analysis was used to identify influential features. **Results**: Among 766 cases, 707 (92.3%) were concordant and 59 (7.7%) were initially discordant. After excluding B3 lesions (*n* = 46), 13 true discordant cases remained (1.7%). Including B3 lesions increased clinically non-concordant or indeterminate cases from 1.7% to 7.7%, indicating that the apparent performance of the models is likely sensitive to case definition and dataset composition. BI-RADS 4a was the most common category (31.3%), and benign pathology (B2) accounted for 64.4% of biopsies. Within this dataset, LightGBM yielded the highest apparent AUC (0.999) (however, given the extremely small number of true discordant cases, this estimate is likely unstable and should be interpreted with caution), while BioBERT showed the strongest agreement with expert consensus (κ = 0.89). SHAP analysis identified clinically meaningful terms such as calcification, hypoechoic, ductal, and carcinoma as key contributors to model predictions. Given the very limited number of true discordant cases, these performance estimates are likely unstable and should be regarded as preliminary, requiring validation in larger, multi-center cohorts. **Conclusions**: This study presents a proof-of-concept NLP-based framework for radiology–pathology concordance assessment. The models showed promising performance in identifying potentially discordant cases; however, given the limited number of true discordant samples, these findings should be considered preliminary and require further validation in larger, multi-center datasets before clinical implementation.

## 1. Introduction

Breast cancer remains the most frequently diagnosed malignancy in women worldwide and a leading cause of cancer-related mortality [1]. Early and accurate diagnosis is critical for guiding treatment and improving outcomes [2]. Radiological imaging—including mammography, ultrasound, and MRI—is standardized via the Breast Imaging Reporting and Data System (BI-RADS), which facilitates structured communication across clinical disciplines [3].

Core needle biopsy or stereotactic biopsy represents the gold standard for tissue sampling in suspicious breast lesions [4,5]. However, diagnostic accuracy depends on careful radiology–pathology correlation. Sampling errors or interpretive discrepancies may lead to false-negative results, making multidisciplinary collaboration essential [4]. Prompt identification of discordance between imaging and pathology findings is crucial to avoid delayed or missed cancer diagnoses [6,7].

Artificial intelligence (AI) and natural language processing (NLP) techniques have shown growing promise in medical imaging, particularly for extracting structured information from unstructured reports. Prior studies have demonstrated successful use of NLP in predicting BI-RADS categories [8], identifying relevant clinical entities [9], and enhancing semantic understanding of radiology texts using transformer-based models [10]. Similar approaches have been applied to pathology reports for staging predictions and prognostic modeling [11,12,13]. Recent reviews emphasize NLP’s potential in radiology reporting, cohort selection, and workflow optimization [14,15,16]. However, despite these advances, no published work has directly implemented NLP-based systems to evaluate radiology–pathology concordance in breast biopsy diagnostics, representing a critical gap in multimodal diagnostic integration. A recent systematic review by Diab et al. highlighted NLP applications in breast imaging reports but did not address intermodality concordance [17].

The primary aim of this study is to explore the feasibility of an NLP-based approach for radiology–pathology concordance assessment in breast biopsy cases. In addition, we aim to investigate whether paired analysis of radiology and pathology reports can support concordance review by identifying cases that may warrant further evaluation. This study is intended as a proof-of-concept framework rather than to establish definitive clinical-grade predictive performance.

## 2. Materials and Methods

### 2.1. Study Design and Setting

This retrospective observational cohort study was conducted at a tertiary referral center specializing in breast imaging and pathology. Ethical approval for this retrospective study was obtained from the ethics committee on 17 September 2025 (Approval No: 17.09.2025.339), and the requirement for informed consent was waived due to the retrospective and anonymized nature of the data. The primary objective was to assess radiology–pathology concordance in breast biopsy cases by leveraging NLP-based methods and machine learning models. Radiology–pathology concordance served as the primary outcome and was established by a multidisciplinary consensus panel comprising two senior breast radiologists and two specialized breast pathologists, representing the diagnostic gold standard. Concordance labels were assigned using predefined radiology–pathology correlation rules based on BI-RADS assessment, pathology category, and final multidisciplinary interpretation. Cases with disagreement were resolved by consensus review between the participating breast radiologists and pathologists. B3 lesions were reviewed separately because they were considered clinically indeterminate rather than straightforwardly concordant or truly discordant. It should be noted that B3 refers to pathology-based classification (lesions of uncertain malignant potential) and should not be confused with BI-RADS 3, which is an imaging-based category.

### 2.2. Patient Selection

All consecutive female patients who underwent breast biopsy between (August 2020–May 2024) were screened. Inclusion criteria were:

1. Availability of a radiology report with BI-RADS categorization;
2. Availability of a corresponding pathology report;
3. Complete clinical documentation in the hospital information system.

Exclusion criteria included incomplete or missing reports, prior history of breast cancer with recurrent lesions, or insufficient follow-up data.

### 2.3. Data Sources and Cohort

A total of 766 breast biopsy cases were included, each consisting of paired radiology and pathology reports, BI-RADS classification, histopathological diagnosis, and final Pathology-B category. Pathology-B classification refers to the standardized histopathological categorization of breast biopsy results, ranging from B1 (normal tissue) to B5 (malignant), and is commonly used in clinical practice to guide management decisions. All data were anonymized in compliance with ethical standards before analysis. Identifiable patient information (e.g., names, ID numbers, physicians, timestamps) was systematically removed.

### 2.4. Data Preprocessing

Unstructured text reports were processed using a multi-stage NLP pipeline designed to handle both Turkish and English representations. All reports were originally written in Turkish. For language-specific preprocessing of pathology reports, Turkish stopwords were removed and lemmatization was performed using the Zeyrek Lemmatizer (version 0.1.3) to preserve clinically meaningful word forms. For downstream modeling requiring a shared semantic representation across report types, both radiology and pathology reports were translated into English using an automated medical translation pipeline. No formal bilingual human validation of the translated texts was performed. Following translation, standard text normalization procedures were applied, including lowercasing, removal of punctuation and non-informative tokens (excluding negation terms such as “not”), and tokenization. For text representation, *n*-gram features (*n* = 1–5) were extracted from radiology reports, while binary bag-of-words (BoW) vectors were generated for pathology reports. In addition to text-derived features, structured clinical variables including lesion size, BI-RADS category, and Pathology-B classification were retained and integrated into the model. To improve confidence in the translation process, a subset of reports (*n* ≈ 20–30) was manually reviewed by a bilingual clinician to assess preservation of key clinical terminology and diagnostic meaning. No major discrepancies affecting clinical interpretation were identified, although minor wording differences were observed. However, no formal quantitative validation (e.g., inter-rater agreement or translation accuracy metrics) was performed, and potential semantic variability introduced by automated translation cannot be fully excluded.

### 2.5. Feature Engineering

Feature construction was based on a combination of structured clinical variables and NLP-derived text representations. Radiology reports were tokenized into *n*-gram features (*n* = 1–5), with binary vectors indicating term presence. Pathology reports were represented using binary bag-of-words (BoW) vectors following language-specific preprocessing.

Structured clinical variables were incorporated alongside text features. BI-RADS categories were encoded as ordinal variables reflecting increasing malignancy risk (2 = 0, 3 = 1, 4a = 2, 4b = 3, 4c = 4, 5 = 5). Pathology-B categories were similarly processed by removing biopsy prefixes (B, C) and applying ordinal encoding (1 = 0, 2 = 1, 3 = 2, 4 = 3, 5a = 4, 5b = 5). Lesion size was retained as a continuous structured variable.

No separate feature selection algorithm was applied; instead, all predefined structured and text-derived features were used as model inputs.

Concordance labels were defined as binary outcomes (concordant = 0, discordant = 1) based on multidisciplinary consensus.

### 2.6. Model Development

Three machine learning models were developed:

1. Decision Tree (baseline): Trained using only categorical BI-RADS and Pathology-B categories.
2. Light Gradient Boosting Machine (LightGBM) (version 4.5.0; Microsoft Corporation, Redmond, WA, USA): Combined categorical inputs with NLP-derived vectors from radiology and pathology reports.
3. Bidirectional Encoder Representations from Transformers pretrained on biomedical corpora (BioBERT): A domain-specific transformer model fine-tuned on preprocessed radiology and pathology texts to capture semantic and contextual nuances.

Given the very low prevalence of true discordant cases, synthetic oversampling techniques (e.g., SMOTE) were not applied, and no alternative imbalance-specific strategies (e.g., class weighting or focal loss) were implemented, as these approaches may introduce artificial patterns that do not reflect real-world clinical distributions. This represents a limitation of the current modeling approach and may affect the model’s ability to robustly detect rare discordant cases.

### 2.7. Training and Evaluation

Data were split into training and testing sets (70:30) using stratified sampling to preserve class distribution. To minimize potential data leakage, each radiology–pathology report pair was treated as an independent sample, and no duplicate or repeated reports from the same clinical encounter were included across the training and test sets. However, patient-level separation between training and test sets could not be fully guaranteed; therefore, some degree of data leakage and performance inflation cannot be excluded. Although efforts were made to avoid duplicate reports, patient-level separation could not be fully guaranteed, which may introduce a potential risk of data leakage. Due to the relatively small number of true discordant cases, special care was taken to maintain class balance during model development. In addition, 5-fold cross-validation was applied to improve robustness and reduce sampling bias. Despite these measures, the limited size of the positive class may affect the stability of performance estimates; therefore, the results should be interpreted as proof-of-concept rather than definitive evidence of generalizable performance. Model hyperparameters were tuned via cross-validation.

Evaluation metrics included:

- Accuracy;
- Sensitivity (Recall);
- Specificity;
- Precision and F1-score;
- Area Under the Receiver Operating Characteristic Curve (AUC);
- Cohen’s Kappa coefficient.

Given the highly imbalanced dataset and the small number of discordant cases, ROC-AUC values may overestimate model performance. Therefore, the reported AUC values should be interpreted as dataset-specific rather than generalizable performance indicators. Discordant cases flagged by the models were retrospectively re-evaluated by expert breast radiologists and pathologists to assess whether additional clinical action (e.g., repeat biopsy or surgical excision) was warranted. This review was performed for interpretative purposes and did not alter the original reference labels.

**Figure 1:**
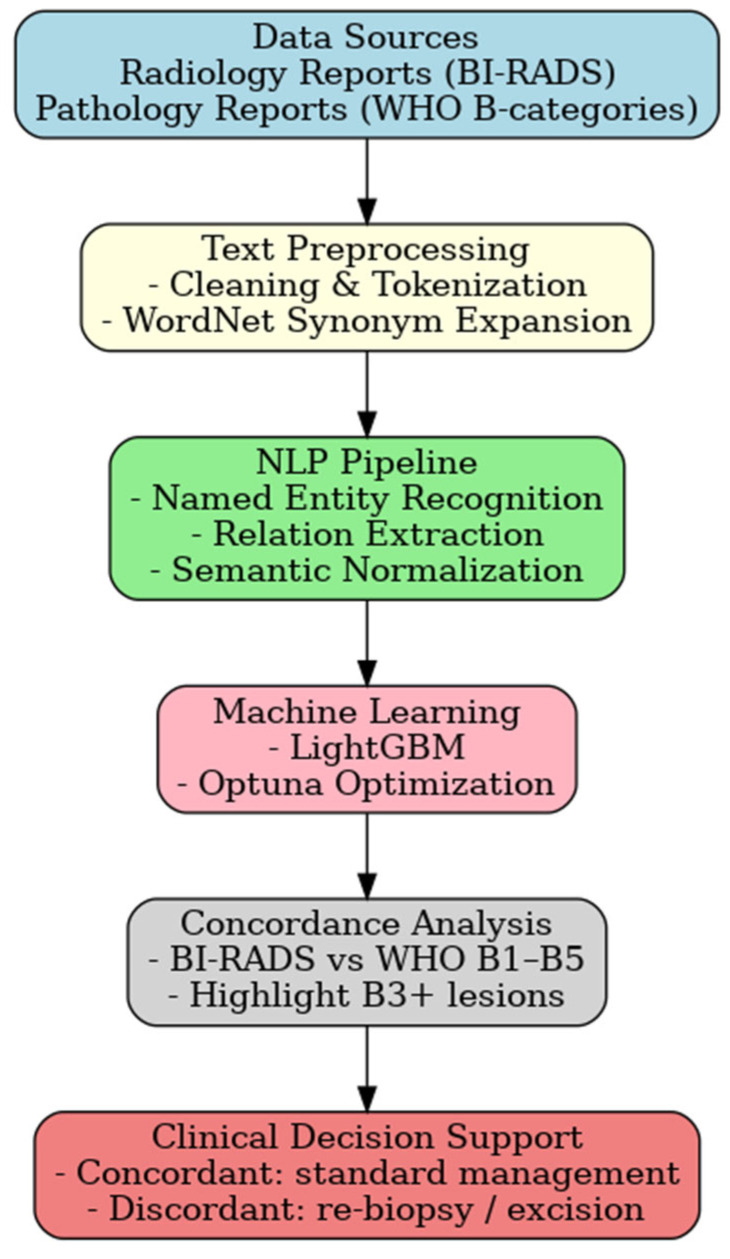
Framework Overview. The following framework chart summarizes the proposed NLP-based radiology–pathology concordance methodology.

The framework chart is summarized in Figure 1. This framework enabled simultaneous semantic alignment of radiology and pathology data, forming the basis for subsequent model training and evaluation. Also, model development and training parameters are presented in Table 1.

### 2.8. Secondary Analytical Approach Including B3 Lesions

In addition to the primary analysis, in which B3 lesions were excluded to focus on clearly concordant versus truly discordant cases, a secondary sensitivity analysis was performed including B3 lesions as clinically indeterminate or potentially non-concordant cases. This approach was adopted to better reflect real-world diagnostic uncertainty and to evaluate the impact of case definition on model performance. The results of this analysis are presented separately.

### 2.9. Statistical Analysis

Statistical analysis was performed to evaluate the performance and reliability of the machine learning models developed for radiology–pathology concordance classification. Descriptive statistics were used to summarize the distribution of BI-RADS categories, pathology B classifications, and concordance status across the study cohort. Categorical variables were presented as frequencies and percentages.

The dataset was randomly divided into training and test sets using a 70:30 split with stratified sampling to preserve the original class distribution, particularly given the low prevalence of true discordant cases. Model performance was assessed using accuracy, sensitivity (recall), specificity, precision, F1-score, area under the receiver operating characteristic curve (AUC), and Cohen’s kappa coefficient to evaluate agreement beyond chance.

Five-fold cross-validation was applied during model training and hyperparameter tuning to minimize overfitting and improve generalizability. Confusion matrices were generated to analyze classification errors and to assess the clinical relevance of false-negative and false-positive predictions. Receiver operating characteristic (ROC) curves were constructed to visualize discriminative performance.

No synthetic oversampling techniques were applied despite class imbalance in order to preserve the natural prevalence of discordant cases. All statistical analyses and model evaluations were performed using Python (version 3.8.0)-based libraries, including scikit-learn, LightGBM, and PyTorch. Generative AI tools were used solely for language editing and improvement of manuscript clarity. No AI tools were used for study design, data collection, analysis, or interpretation.

## 3. Results

A total of 766 breast biopsy cases were included in the study. Of these, 707 cases (92.3%) demonstrated radiology–pathology concordance, while 59 cases (7.7%) were initially classified as discordant. Among these, 46 cases (6.0%) corresponded to B3 lesions, representing entities of uncertain malignant potential. These cases were not considered true discordances but rather indeterminate findings requiring further clinical management, such as repeat biopsy or surgical excision. After excluding B3 lesions, 13 cases (1.7%) were confirmed as true radiology–pathology discordant cases. Given the clinical heterogeneity of B3 lesions, subtype analyses were performed across Atypical Ductal Hyperplasia (ADH), Flat Epithelial Atypia (FEA), Lobular Neoplasia (LIN), papillary lesions, and radial scar. Detailed results for model sensitivity and upgrade risk by B3 subtype are presented in Table 2.

The distribution of biopsy cases across BI-RADS categories is presented in Table 3. The largest proportion of lesions were classified as BI-RADS 4a (240 cases, 31.3%), followed by BI-RADS 3 (213 cases, 27.8%). Histopathological outcomes are also summarized in Table 3. The majority of biopsies yielded benign results (B2: 493 cases, 64.4%), while invasive carcinoma (B5b: 183 cases, 23.9%) was the second most frequent diagnosis. Indeterminate lesions (B3: 46 cases, 6.0%) represented a smaller yet clinically significant subset of the cohort. Overall, the distribution of BI-RADS and pathology B categories was consistent with the expected clinical spectrum, with the highest representation in BI-RADS 4a and Pathology B2. The cross-tabulation of BI-RADS categories with pathology B classifications, along with concordance status, is summarized in Table 3. After excluding benign (B2) lesions, the dataset comprised 273 non-benign breast biopsy cases. The adjusted concordance rate decreased to 78.4%, while discordant or indeterminate (B3) findings accounted for 21.6% of all cases. True discordant cases represented 4.8% of the total, reflecting the clinically meaningful discordance rate among higher-risk biopsies.

### 3.1. Word Frequency Analysis of Radiology and Pathology Reports

**Figure 2:**
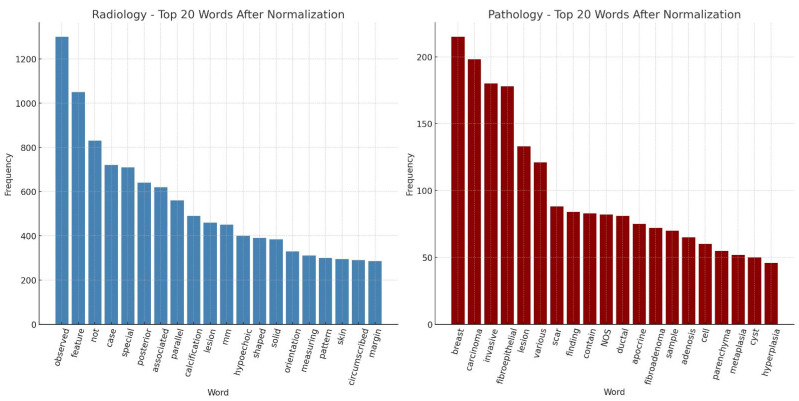
The graphical illustration of the top 20 most frequent words in both radiology and pathology reports.

**Figure 3:**
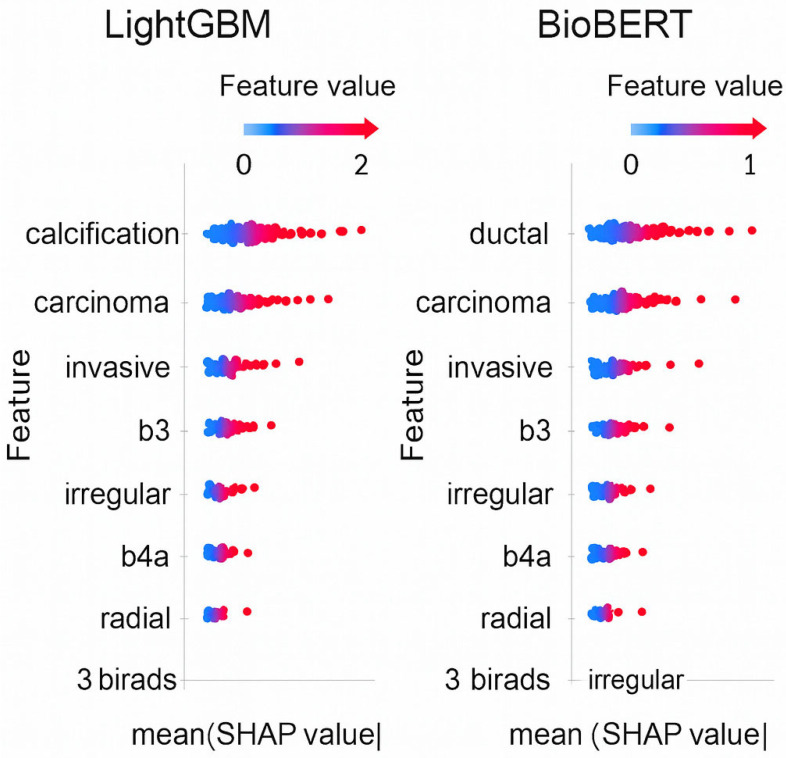
SHAP analysis summary plots for the LightGBM and BioBERT models, showing the most influential radiologic and pathologic features (e.g., *calcification*, *hypoechoic*, *ductal*, *carcinoma*, *fibroepithelial*) contributing to concordance or discordance predictions.

Following normalization, we analyzed the top 20 most frequent words in both radiology and pathology reports (Figure 2). In radiology texts, commonly occurring descriptors included “observed,” “feature,” “posterior,” “calcification,” “lesion,” and “hypoechoic,” reflecting BI-RADS–related terminology for lesion characterization. In pathology reports, the most frequent terms were “breast,” “carcinoma,” “invasive,” “fibroepithelial,” “ductal,” and “fibroadenoma,” highlighting the histopathological spectrum of breast biopsies. Comparing these top 20 words demonstrates how radiology emphasizes imaging-based morphological features, while pathology captures definitive diagnostic entities. This complementary distribution underscores the importance of NLP-based normalization to harmonize vocabularies across modalities, ensuring semantic alignment and enabling robust radiology–pathology concordance modeling. Additionally, to ensure model transparency, SHapley Additive exPlanations (SHAP) analyses were conducted for LightGBM and BioBERT. Top contributing features included: *“calcification,” “hypoechoic,” “ductal,” “carcinoma,” “fibroepithelial.”* SHAP analysis of LightGBM and BioBERT was shown in Figure 3. These findings suggest that the models rely on clinically meaningful terms related to lesion characterization and malignancy, supporting the interpretability of the NLP-based approach.

### 3.2. Model Performance Summary

The confusion matrix-based evaluation demonstrated favorable apparent performance metrics in this dataset across all machine learning models. BioBERT, LightGBM, and Decision Tree classifiers each achieved accuracy rates above 95%. BioBERT provided the best balance between sensitivity and specificity, while LightGBM exhibited the highest sensitivity in detecting discordant cases. The Decision Tree model, although slightly less precise, still performed within clinically acceptable diagnostic thresholds. Comprehensive performance metrics, including accuracy, sensitivity, specificity, precision, recall, F1-score, and Cohen’s Kappa, are summarized in Table 4. BioBERT achieved the highest Cohen’s Kappa, reflecting robust agreement beyond chance between predicted and ground-truth labels. LightGBM showed the highest sensitivity, reinforcing its utility in detecting discordant cases. Decision Tree, while less optimal in precision, maintained moderate performance across all parameters. Despite the favorable apparent performance metrics observed across models, these results should be interpreted cautiously, as the very limited number of true discordant cases may lead to optimistic and potentially unstable estimates. Also, comparative model performance after excluding benign (B2) cases is presented in Table 5. After exclusion, LightGBM demonstrated the highest sensitivity (97.0%) and AUC (0.982), indicating superior capability in detecting discordant cases. BioBERT maintained the strongest semantic agreement (Kappa = 0.89), while Decision Tree provided interpretable rule-based decisions within acceptable diagnostic performance thresholds.

### 3.3. ROC Curve Analysis

**Figure 4:**
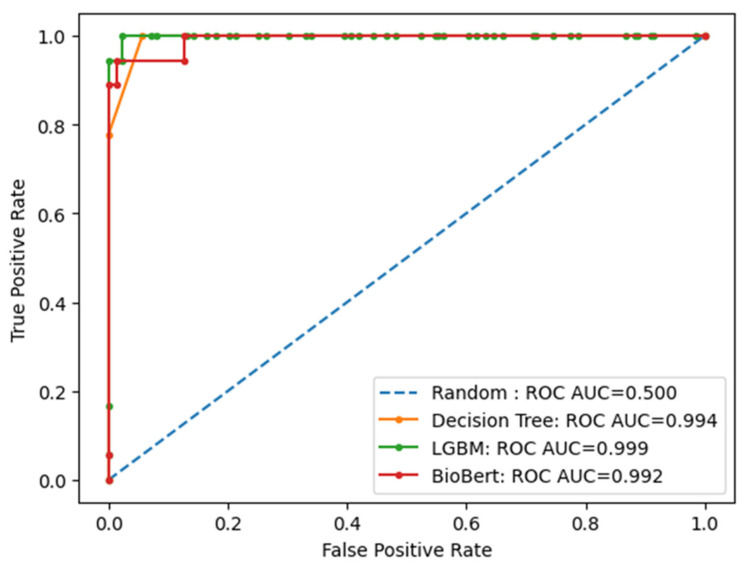
Receiver Operating Characteristic (ROC) curves for the BioBERT, LightGBM, and Decision Tree models for concordance classification. All models demonstrated apparent discriminative performance within this dataset, with LightGBM showing the highest AUC.

Receiver Operating Characteristic (ROC) curve analysis suggested apparent class separation within this dataset. All classifiers achieved AUC values greater than 0.95, with LightGBM consistently demonstrating the highest discriminative ability (AUC = 0.999), followed by Decision Tree (AUC = 0.994) and BioBERT (AUC = 0.992) (Figure 4). These results highlight the superior performance of LightGBM in distinguishing concordant from discordant cases. The near-perfect AUC values (approaching 1.0) are likely influenced by the small number of positive (discordant) cases, which can artificially inflate ROC-based performance metrics.

### 3.4. Sensitivity Analysis Including B3 Lesions

When B3 lesions are included, the number of clinically non-concordant or indeterminate cases increases from 13 (1.7%) to 59 (7.7%), reflecting a more realistic representation of diagnostic uncertainty in routine clinical practice. Under this expanded definition, the dataset becomes more heterogeneous, incorporating borderline and ambiguous cases commonly encountered in clinical workflows. In the primary analysis excluding B3 lesions, all models demonstrated very high apparent performance, with AUC values approaching 1.0. However, when B3 lesions were included, AUC values decreased across all models, indicating reduced discriminative performance in a more heterogeneous and clinically realistic dataset. This finding suggests that the near-perfect performance observed in the primary analysis may be partly driven by the exclusion of borderline cases and highlights that model performance is sensitive to case definition and dataset composition.

## 4. Discussion

Radiology–pathology concordance is a critical quality control checkpoint in breast cancer diagnostics, as mismatches between imaging findings and histopathology may lead to delayed or inappropriate clinical management. Although traditional workflows rely on manual consensus between radiologists and pathologists, studies show that discordant results—particularly in BI-RADS 4 and 5 lesions—can harbor malignancy despite an initially benign pathology report [18]. In a large cohort study, Wan et al. analyzed 3080 core needle biopsies and found that 2.1% of cases initially deemed benign were actually discordant upon further investigation [19]. Importantly, 30.9% of these discordant lesions were eventually diagnosed as malignant. These findings underscore the importance of identifying such cases for further clinical evaluation.

Management of B3 lesions—those of uncertain malignant potential—remains a key diagnostic challenge. According to the WHO classification and large meta-analyses, B3 lesions demonstrate an overall malignancy upgrade rate ranging from 17% to 35%, depending on the histologic subtype [20]. Willers et al. reported upgrade rates of 19.0% for B3 lesions diagnosed via core needle biopsy and 14.9% for those sampled with vacuum-assisted biopsy [21]. In this context, treating B3 lesions as indeterminate rather than definitively concordant aligns with clinical practice and supports cautious follow-up strategies. Delayed recognition of such lesions or radiology–pathology mismatch has been associated with delayed cancer diagnosis. Early identification of such cases may facilitate timely additional imaging, re-biopsy, or surgical intervention. This approach is consistent with guideline recommendations emphasizing multidisciplinary evaluation and shared decision-making for lesions of uncertain malignant potential [22,23,24]. Emerging literature on AI in imaging and digital pathology also suggests potential improvements in diagnostic consistency and reduction in variability, which may contribute to better patient outcomes and optimized resource utilization [25,26,27,28]. The observed performance is strongly influenced by the operational definition of discordance. Exclusion of B3 lesions reduces clinical ambiguity and simplifies the classification task, likely contributing to the near-perfect performance observed in this dataset, particularly given the very small number of true discordant cases. When B3 lesions are included, the dataset becomes more heterogeneous and better reflects real-world diagnostic uncertainty, highlighting that concordance is not strictly binary in clinical practice. Therefore, the findings should be interpreted as dataset-specific and exploratory rather than indicative of generalizable predictive capability.

While radiology–pathology concordance has traditionally been assessed manually, recent studies have applied NLP techniques primarily to radiology reports. Bozkurt et al. developed an NLP model to extract BI-RADS descriptors from mammography reports with high classification accuracy [8], and Kuling et al. applied BERT-based architectures to improve semantic representation of radiology texts [10]. However, these approaches do not incorporate pathology data or explicitly model concordance relationships. In contrast, our study integrates both radiology and pathology reports through parallel NLP pipelines, enabling concordance-aware analysis. Among the evaluated models, BioBERT achieved the best overall balance between sensitivity and specificity, while LightGBM showed the highest sensitivity and discriminative performance within this dataset. However, given the small number of true discordant cases, these findings should be interpreted cautiously as preliminary. The Decision Tree model, although less precise, provided interpretable predictions that may support transparency in clinical settings. In addition, the use of bag-of-words representations in classical models may limit the ability to capture contextual semantics compared with transformer-based approaches, representing an area for future improvement. When B2 (benign) lesions were excluded, model performance improved, particularly in sensitivity and AUC, suggesting that concordance assessment may be more discriminative in higher-risk subsets. These findings align with the limited literature highlighting the potential of NLP in breast imaging workflows while emphasizing the gap in concordance-focused applications [17]. Thus, our work represents a methodological advancement in the field—moving from passive data structuring toward decision-support for concordance review through concordance-aware AI pipelines.

These findings suggest that NLP-based approaches may support structured concordance review by identifying cases that warrant further evaluation. Such tools may be particularly useful in high-volume settings or institutions without specialized breast imaging expertise, where consistent report interpretation may be challenging. Rather than replacing multidisciplinary review, the proposed system is intended to function as a supportive, report-level decision aid that helps prioritize cases for closer examination.

To our knowledge, this is the first study to apply NLP and deep learning models for concordance-aware analysis of paired radiology and pathology reports in breast biopsy. Nevertheless, this study has several important limitations. A major limitation is the extremely small number of true radiology–pathology discordant cases (*n* = 13), which limits the statistical reliability of model evaluation. In such settings, performance metrics such as ROC-AUC may be overly optimistic and sensitive to small variations in the data, potentially leading to inflated performance estimates. Precision–recall-based evaluation would be more appropriate in the context of severe class imbalance and should be considered in future work. Furthermore, the absence of external validation and the lack of dedicated strategies to address class imbalance (e.g., resampling or cost-sensitive learning) further limit the generalizability of the findings. Precision–recall analysis would provide a more appropriate evaluation in the context of class imbalance and should be considered in future work. Accordingly, these results should be considered preliminary and require validation in larger and more diverse cohorts. In addition, the single-center and single-language nature of the dataset further restricts generalizability. All reports were generated within one institution and originally written in Turkish, then translated into English for NLP. The absence of formal bilingual validation may introduce semantic bias. Moreover, the model is based exclusively on textual data and does not incorporate imaging or histopathological slide-level information; therefore, factors such as lesion targeting accuracy and sampling adequacy cannot be assessed. Finally, the clinical workflow impact of the system was not prospectively evaluated, and its potential benefits remain hypothetical. Future studies should include multicenter validation, multilingual datasets, and prospective implementation to assess real-world applicability.

## 5. Conclusions

This study demonstrates that radiology–pathology discordance remains a relevant challenge in breast cancer diagnostics, and that automated, NLP-based concordance assessment tools may help improve diagnostic quality, especially in complex or ambiguous cases. We developed a novel NLP-based system that showed promising performance in detecting radiology–pathology discordance in breast biopsy cases. By integrating structured imaging and pathology data with contextual language understanding, the model may support concordance review and assist in identifying cases that warrant additional evaluation. Unlike previous NLP tools focused solely on report extraction, our approach may assist in identifying cases that warrant multidisciplinary reassessment. These findings highlight the potential of AI-assisted concordance analysis to support clinical review processes, pending further validation.

## Figures and Tables

**Table 1:** Comparison of model characteristics and input features for the Decision Tree, LightGBM, and BioBERT models used in radiology–pathology concordance assessment.

Feature	Decision Tree	LightGBM	BioBERT
Model type	Tree-based classifier	Gradient boosting model	Transformer-based model
Input data	Structured variables	Structured + text features	Full-text reports
Text representation	Ordinal encoding	*n*-grams + Bag-of-Words	Contextual embeddings
Structured variables	Included	Included	Included (metadata context)
Feature integration	Structured only	Combined structured + text	Text-based representation
Interpretability	High	Moderate	Low
Implementation	scikit-learn (version 1.2.2)	LightGBM (version 4.5.0)	PyTorch (version 2.4.1) (BioBERT)

**Table 2:** Distribution of B3 lesion subtypes and associated upgrade risk following breast biopsy.

B3 Subtype	*n*	Literature Upgrade Rate (%)	Model Sensitivity (%)	Notes
ADH (Atypical Ductal Hyperplasia)	18	22–35%	96.4%	Highest upgrade potential; model performs strongly
FEA (Flat Epithelial Atypia)	9	17–25%	94.1%	Frequently upgraded when associated with calcifications
LIN/ALH (Lobular Neoplasia)	7	18–30%	88.7%	Lower sensitivity due to subtle radiologic correlates
Papillary Lesions	8	10–20%	91.5%	Model captures descriptive terms effectively
Radial Scar/Complex Sclerosing Lesion	4	8–17%	89.3%	Small sample size but performance stable

**Table 3:** Cross-tabulation of BI-RADS categories and pathology B classifications with radiology–pathology concordance status.

BI-RADS Category	Pathology B Category	Concordant (*n*)	Discordant (*n*)	Total (*n*)	Concordant (%)	Discordant (%)	Total (%)
2	2	43	0	43	5.8	0.0	5.8
2	3	0	1	1	0.0	0.1	0.1
2	4	0	2	2	0.0	0.3	0.3
3	2	203	0	203	27.3	0.0	27.3
3	3	0	10	10	0.0	1.3	1.3
4a	1	0	15	15	0.0	2.0	2.0
4a	2	194	0	194	25.3	0.0	25.3
4a	3	25	0	25	3.3	0.0	3.3
4a	4	1	0	1	0.1	0.0	0.1
4a	5b	5	0	5	0.7	0.0	0.7
4b	1	0	7	7	0.0	0.9	0.9
4b	2	32	14	46	4.2	1.8	6.0
4b	3	10	0	10	1.3	0.0	1.3
4b	4	2	0	2	0.3	0.0	0.3
4b	5a	6	0	6	0.8	0.0	0.8
4b	5b	25	0	25	3.3	0.0	3.3
4c	1	0	3	3	0.0	0.4	0.4
4c	2	0	5	5	0.0	0.7	0.7
4c	5a	5	0	5	0.7	0.0	0.7
4c	5b	40	0	40	5.3	0.0	5.3
5	2	0	2	2	0.0	0.3	0.3
5	5a	3	3	6	0.4	0.4	0.8
5	5b	113	0	113	15.0	0.0	15.0
Total	-	707	59	766	92.3	7.7	100.0

This table illustrates the relationship between imaging suspicion and histopathologic outcomes, and identifies patterns associated with discordant or indeterminate diagnoses.

**Table 4:** Comparative Performance Metrics of Machine Learning Models for Radiology–Pathology Concordance Classification.

Metric	Decision Tree	LightGBM	BioBERT
Accuracy (%)	95.1	97.8	96.9
Sensitivity (Recall)	93.0	98.6	95.4
Specificity	96.2	97.1	97.5
Precision	92.7	94.8	96.0
F1-Score	92.8	96.6	95.7
AUC (ROC)	0.994	0.999	0.992
Cohen’s Kappa	0.87	0.90	0.92

Notes: LightGBM showed the highest apparent ROC-based performance within this dataset (AUC = 0.999); however, this estimate should be interpreted cautiously given the limited number of discordant cases. BioBERT achieved the strongest inter-rater agreement (Cohen’s Kappa = 0.92), reflecting good alignment with expert consensus. The Decision Tree model provided interpretable results, though with slightly lower precision and recall, remaining within acceptable analytical performance ranges.

**Table 5:** Performance of Machine Learning Models After Excluding Benign (B2) Cases.

Model	Accuracy	Sensitivity	Specificity	AUC	Kappa
Decision Tree	93.5%	90.1%	94.3%	0.95	0.83
LightGBM	96.8%	97.0%	96.0%	0.982	0.88
BioBERT	95.6%	91.2%	97.4%	0.978	0.89

Legend: Comparative model performance after excluding benign (B2) cases. LightGBM demonstrated the highest sensitivity (97.0%) and AUC (0.982), indicating superior capability in detecting discordant cases. BioBERT maintained the strongest semantic agreement (Kappa = 0.89), while Decision Tree provided interpretable rule-based decisions within acceptable diagnostic performance thresholds.

## Data Availability

The datasets generated and analyzed during the current study are not publicly available due to institutional privacy restrictions but are available from the corresponding author upon reasonable request. Due to institutional and privacy constraints, the dataset cannot be publicly shared; however, model code and preprocessing pipelines can be made available upon reasonable request.

## Abbreviations

BIRADS (Breast Imaging Reporting and Data System), AI (Artificial intelligence), NLP (Natural language processing), LightGBM (Light Gradient Boosting Machine), AUC (Area Under the Curve), ROC (Receiver Operating Characteristic).
